# The Structure of the Red Blood Corpuscle

**Published:** 1913-10-25

**Authors:** 


### The Structure of the Red Blood Corpuscle.

In the *Medical Chronicle* Dr. E. M. Brockbank records some exceedingly interesting observations which throw light upon the cell structure of the red corpuscle. Exactly what bearing these observations have upon the many different kinds of anæmia is not yet clear; but further investigations upon the same lines may easily prove very fruitful in clarifying our conceptions of these diseases. Dr. Brockbank submits that his experiments show: That when a red corpuscle is subjected to the action of pepsin in very weak acid solution some change takes place as the result of peptic digestion, which renders the corpuscle very resistant to other and more marked changes which take place at once when the acidity of the pepsin solution is increased. That when the acidity of the pepsin solution is a degree greater, the red corpuscle is distended either entirely or locally in a remarkable manner, and the hæmoglobin goes into solution; in this process of its removal the hæmoglobin may be combined with lecithin. That when the acidity of the pepsin solution is still greater (about .01 per cent.) the hæmoglobin is removed from the corpuscle by a process which is visible under the microscope, leaving the remains of the envelope of the corpuscle as a shell or "shadow," this shell being unstainable by ordinary blood dyes. From these Dr. Brockbank derives a conception of the structure of the red blood corpuscle as follows: The outer layer is a structureless protecting material; inside this is the true envelope of the corpuscle, which has no histological structure, but is capable of coalescing with similar material from other corpuscles to form a bigger mass, or of dividing into smaller masses (like a soap bubble). Within the true envelope is the hæmoglobin, which is probably held by some uncertain body linking it to the true envelope and not merely held in watery solution. It is possible that this connecting substance is lecithin. Within the envelope hæmoglobin capsule, in some conditions, is a common cavity, not a spongework as most physiologists have supposed, containing the material which when extruded forms blood platelets.

